# Mapping Resident Competency: National Survey on Geriatric Milestones in Medication Management, Behavioral Health, Substance Use Disorders, and Patient Safety by the LIGHTS-Rx Harm Reduction Program

**DOI:** 10.51894/001c.144192

**Published:** 2025-09-30

**Authors:** Monica Pelowski

**Affiliations:** 1 College of Osteopathic Medicine Michigan State University

## 52

### INTRODUCTION

The growing demand for competent geriatric care highlights the need for effective training of healthcare residents in key areas. With a shortage of geriatric psychiatrists to address the needs of the aging population, primary care providers are increasingly tasked with managing behavioral, affective, and substance use issues in older adults. Early data suggests gaps in training that may hinder the ability of residents to deliver optimal care. In response, the Limiting Inpatient Geriatric Hospitalizations through Safer Prescribing and Practice (LIGHTS-Rx) program was developed to enhance resident training and improve health literacy in older adults.

### OBJECTIVES

This study aims to assess residents’ self-reported competencies in geriatric care across multiple specialties, focusing on medication management, behavioral health assessment, patient safety, and substance use disorder management. The study also seeks to identify training gaps and explore resident experiences to guide curricular improvements.

### METHODS

A nationwide survey was developed based on the American Geriatrics Society’s residency competency milestones and supplemented by guidelines from the Substance Abuse and Mental Health Services Administration. Residency program contact information was sourced from the FREIDA™ database for survey distribution. Data were collected via Qualtrics and Google Forms and analyzed using SPSS with descriptive statistics, ANOVA, and Chi-Square tests.

### RESULTS

Data collection is ongoing, with completion expected by Summer 2025. Preliminary findings indicate significant gaps in self-perceived competencies, particularly in the areas of delirium, dementia, and deprescribing. Additionally, there is strong consensus among residents that training in substance use disorder management is inadequate.

### CONCLUSIONS

This national survey underscores critical gaps in resident training in geriatric care. The findings will inform the development of educational strategies aimed at enhancing residency curricula, improving resident competency, and ultimately enhancing patient outcomes for this vulnerable population.

